# Nonlinear effects of environmental drivers shape macroinvertebrate biodiversity in an agricultural pondscape

**DOI:** 10.1002/ece3.9458

**Published:** 2022-11-08

**Authors:** Camille L. Musseau, Gabriela Onandia, Jana S. Petermann, Alban Sagouis, Gunnar Lischeid, Jonathan M. Jeschke

**Affiliations:** ^1^ Institute of Biology Freie Universität Berlin Berlin Germany; ^2^ Berlin‐Brandenburg Institute of Advanced Biodiversity Research Berlin Germany; ^3^ Leibniz Institute of Freshwater Ecology and Inland Fisheries (IGB) Berlin Germany; ^4^ Leibniz Centre for Agricultural Landscape Research (ZALF) Müncheberg Germany; ^5^ Department of Environment and Biodiversity University of Salzburg Salzburg Austria; ^6^ German Centre for Integrative Biodiversity Research (iDiv) Halle‐Jena‐Leipzig Leipzig Germany; ^7^ Department of Computer Science Martin Luther University, Halle‐Wittenberg Halle Germany; ^8^ Institute for Environmental Sciences and Geography University of Potsdam Potsdam Germany

**Keywords:** agriculture, benthic invertebrates, eutrophication, kettle holes, rural ponds

## Abstract

Agriculture is a leading cause of biodiversity loss and significantly impacts freshwater biodiversity through many stressors acting locally and on the landscape scale. The individual effects of these numerous stressors are often difficult to disentangle and quantify, as they might have nonlinear impacts on biodiversity. Within agroecosystems, ponds are biodiversity hotspots providing habitat for many freshwater species and resting or feeding places for terrestrial organisms. Ponds are strongly influenced by their terrestrial surroundings, and understanding the determinants of biodiversity in agricultural landscapes remains difficult but crucial for improving conservation policies and actions. We aimed to identify the main effects of environmental and spatial variables on α‐, β‐, and γ‐diversities of macroinvertebrate communities inhabiting ponds (*n* = 42) in an agricultural landscape in the Northeast Germany, and to quantify the respective roles of taxonomic turnover and nestedness in the pondscape. We disentangled the nonlinear effects of a wide range of environmental and spatial variables on macroinvertebrate α‐ and β‐biodiversity. Our results show that α‐diversity is impaired by eutrophication (phosphate and nitrogen) and that overshaded ponds support impoverished macroinvertebrate biota. The share of arable land in the ponds' surroundings decreases β‐diversity (i.e., dissimilarity in community), while β‐diversity is higher in shallower ponds. Moreover, we found that β‐diversity is mainly driven by taxonomic turnover and that ponds embedded in arable fields support local and regional diversity. Our findings highlight the importance of such ponds for supporting biodiversity, identify the main stressors related to human activities (eutrophication), and emphasize the need for a large number of ponds in the landscape to conserve biodiversity. Small freshwater systems in agricultural landscapes challenge us to compromise between human demands and nature conservation worldwide. Identifying and quantifying the effects of environmental variables on biodiversity inhabiting those ecosystems can help address threats impacting freshwater life with more effective management of pondscapes.

## Graphical abstract

Working on 42 small freshwater ponds, our results show that: (1) α‐diversity is impaired by eutrophication (phosphate and nitrogen) and that overshaded ponds support impoverished macroinvertebrate biota; (2) the share of arable land in the ponds' surroundings decreases β‐diversity (i.e., dissimilarity in community composition among sites), while β‐diversity is higher in shallower ponds; and (3) β‐diversity is mostly driven by taxonomic turnover and that ponds embedded in arable fields support local and regional diversity. This highlights the importance of such ponds for supporting biodiversity, identify the main stressors related to human activities (eutrophication), and emphasize the need for a large number of ponds in the landscape to conserve biodiversity.

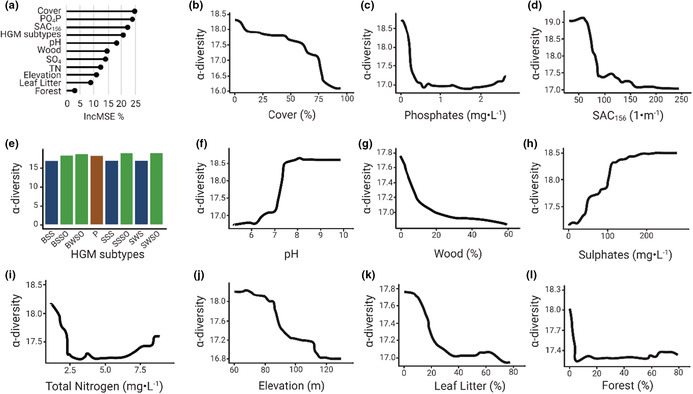

## 1 INTRODUCTION

A third of the world's landmass has been converted to agriculture, leading to the destruction and fragmentation of the remaining natural habitats, and driving the decline of biodiversity (IPBES, 2019). Agriculture intensification and industrialization have substantially increased fertilizers and other chemical inputs, dramatically impacting biodiversity and ecosystem functioning (Stehle & Schulz, 2015; Wolfram et al., 2021). Freshwater ecosystems are significantly affected by land‐use and agriculture‐related interacting stressors, reducing freshwater biodiversity through habitat degradation, eutrophication, and diverse, diffuse pollutions (Birk et al., 2020; Dudgeon et al., 2006; Reid et al., 2019). However, the individual effects of these numerous stressors are often difficult to disentangle and quantify, as they might have nonlinear impacts on biodiversity and recipient ecosystems (Birk et al., 2020; Ormerod et al., 2010). Consequently, understanding the determinants of freshwater biodiversity in agricultural landscapes remains a difficult but necessary task for improving conservation policies.

Agriculture and multiple‐related stressors can modify the spatial distribution of species due to dispersal limitations and niche processes (Jeliazkov et al., 2016; Onandia et al., 2021). Therefore, land‐use intensity and types can play a role in driving species assemblages living in individual habitats (α‐diversity), the assemblages' differentiation among sites (β‐diversity), and the species pool of a landscape (γ‐diversity). Overall, freshwater biodiversity responses to agriculture depend on the scale, taxa, and stressors considered: α‐, β‐, and γ‐diversities can increase (Fugère et al., 2016), decrease (Rosset et al., 2014; Siqueira et al., 2015), or remain unchanged in response to agriculture (Rosset et al., 2014; Socolar et al., 2016). Therefore, understanding and predicting the impacts of intensive agriculture has remained challenging. Partitioning β‐diversity and quantifying the respective roles of species replacement (turnover) and species loss/gain (nestedness) is essential to understand the causal mechanisms structuring biodiversity in ecosystems embedded in agricultural landscapes for improving conservation strategies (Baselga, 2010; Hill et al., 2017).

Small freshwater bodies (i.e., ponds, ditches, streams) are widely distributed in agricultural landscapes and form an essential part of the continental freshwater resources. Ponds, defined as small lentic water bodies (<2 ha in area, Biggs et al., 2005), represent up to 30% of the global standing freshwater per surface area and 90% of the global standing water bodies (Downing et al., 2009). Strongly influenced by their terrestrial surroundings, ponds are threatened by an extensive range of stressors in agricultural landscapes (Usio et al., 2017). Surface runoff can result in excess nutrients, such as nitrogen and phosphorus, which are highly amended in intensively exploited crops leading to eutrophication (Guignard et al., 2017). Ponds also receive large amounts of terrestrial‐derived organic matter, particularly from riparian vegetation which may strongly affect biodiversity (Bartels et al., 2012). The impacts of agriculture on riparian vegetation thus also indirectly shape the biodiversity of small freshwater bodies (Fierro et al., 2017; Hykel et al., 2016).

Although interest in the biodiversity of ponds and pondscapes (i.e., networks of ponds and surrounding terrestrial matrix, Hill et al., 2018) has grown during the last decade (Céréghino et al., 2014), these ecosystems are still understudied compared to the larger freshwater systems such as rivers and lakes (Hill et al., 2021). Notably, ponds in agricultural landscapes support a higher number of species than rivers, streams, and ditches (Williams et al., 2004). In homogenized environments such as agroecosystems in which large arable fields dominate the landscape, ponds may be biodiversity hotspots significantly contributing to freshwater biodiversity conservation and ecosystem functioning (Biggs et al., 2017). They provide suitable habitats for a wide range of freshwater species, including macrophytes (Lozada‐Gobilard et al., 2019), zooplankton (Onandia et al., 2021), macroinvertebrates (Hill et al., 2016), and vertebrates (Knutson et al., 2004). Ponds are also essential for semi‐aquatic and terrestrial species and play a crucial role in food‐web dynamics, with emerging adult aquatic insects linking freshwater and terrestrial food webs by transporting aquatic subsidies towards terrestrial ecosystems and representing a substantial source of energy for terrestrial predators [e.g., bats (Heim et al., 2018), birds (Lewis‐Phillips et al., 2020), and carabids (Batzer & Wu, 2020)]. Due to human activities, the diversity of macroinvertebrates—including insects—experiences a substantial decline worldwide (Sánchez‐Bayo & Wyckhuys, 2019), which significantly impairs freshwater ecosystem functioning (Cao et al., 2018). Understanding the consequences of land‐use on macroinvertebrate biodiversity in modified landscapes is crucial for implementing conservation measures to protect freshwater biodiversity.

In the present study, we focus on macroinvertebrate communities in a network of shallow ponds (kettle holes) located in an intensively used landscape consisting of arable fields and patches of grasslands and forests in the North‐eastern Germany. Those ponds exhibit high natural environmental variations in physical–chemical properties, canopy cover, hydroperiod, and hydrogeomorphic subtypes that can relate to the ecological gradients in stages of pond succession (Kalettka & Rudat, 2006). They also collect inputs from anthropogenic activities derived from agricultural practices (nutrients, etc., Nitzsche et al., 2017), depending on the land‐use categories and types of crops in the surroundings. Even if biodiversity has been homogeneised in this landscape (Ionescu et al., 2022), pond communities continue to respond to agriculture and environmental variation (Bižić et al., 2022). Here, we aimed to: (1) quantify α‐, β‐, and γ‐diversities of macroinvertebrates inhabiting ponds embedded in the different land‐use types, (2) identify the main variables shaping α‐ and β‐diversities and quantify their main effects, and (3) disentangle the respective roles of spatial turnover and nestedness in β‐diversity among land‐use categories and types of crops.

## 2 MATERIAL AND METHODS

### 2.1 Study area

The study was conducted in spring 2017 in the Uckermark region (North Brandenburg, Germany). This area is characterized by a continental climate with an average annual temperature above 9°C. Among the driest regions in Germany, the average annual rainfall is 514 mm (1981–2010), and precipitation reached 459 mm in the study year (Station Angermünde, Uckermark, Germany, DWD 2020).

The study region covers a 220 km^2^ area within a young moraine landscape that has been shaped by glacier activity during the last ice age. This area has long been used for agricultural activities, and the landscape has been modified many times for increasing field size (average size for arable fields: 50 ha, up to 200 ha), soil yield, and food production (Kleeberg et al., 2016). Nowadays, those natural shallow freshwater bodies are in a highly modified landscape dominated by intensive agriculture (75% of the surface area) with patches of grasslands and forests.

We sampled 42 ponds for macroinvertebrate communities—these included 29 ponds in arable fields, five in grasslands, and eight in forests; most of them were fishless. Ponds embedded in arable fields were surrounded by different crops (barley, corn, rapeseed, and wheat). The grasslands were mostly used for hay production, and the forests were dominated by native deciduous tree species, mainly European beech (*Fagus sylvatica*) and birch (*Betula* spp.).

### 2.2 Sampling

Each pond was sampled once for macroinvertebrates between May 31 and June 21, 2017. Macroinvertebrates were collected using a pond dipping net (width: 25 cm; mesh size: 250 μm) in the different habitats (i.e., surface sediment, dense vegetation, other plant material, see Table 1). Each pond dipping was done on a 1‐m transect and took between 30 and 40 s, consisting of the intensive sweeping of the net through the habitat. The action was carefully repeated three times to collect the invertebrates swept away. The number of sampled transects ranged from 3 to 7 based on pond surface area (Dryad dataset, https://doi.org/10.5061/dryad.fj6q573zf). Invertebrate samples were preserved in the field in 70% ethanol and brought to the lab, where they were identified at the genus level, except Diptera and Oligochaeta which were identified at the family level. To standardize effort among the ponds, total abundances per pond were divided by the number of transects. To standardize for differences in levels of taxonomic identification between groups, the following analyses were performed at two identification levels: (1) the *taxonomic richness* referring to the number of Diptera and Oligochaeta families plus the number of genera for the rest of the groups, and (2) the *family richness* referring to the number of families for the different organisms identified in the samples.

**TABLE 1:** Spatial and environmental variables measured and used in the present study

Group	Variable	Unit	Description
Spatial	Latitude	°	North–south position
	Longitude	°	East–west position
	Elevation	m	Height above sea level
	Closest pond	m	Distance to the closest pond; proxy for dispersal and colonization abilities
	Pond density	−	Number of ponds in the surroundings (1 km buffer); proxy for dispersal and colonization abilities
Land‐use	Arable land	%	Arable land in a buffer of 1 km from the pond's centre
	Grasslands	%	Grassland area in a buffer of 1 km from the pond's centre
	Forests	%	Forest area in a buffer of 1 km from the pond's centre
	Sealed land	%	Area belonging to farms and roads in a buffer of 1 km from the pond's centre
	Land‐use categories	−	Categories of land‐use in which a pond is embedded: arable fields, grasslands, forests
	Crops	−	barley, corn, rapeseed, wheat
Habitat	Surface area	ha	Surface area of the pond
	Depth	cm	Mean depth sampled
	Wood	%	Transect area covered by woody substrate
	Roots	%	Transect area covered by roots
	Leaf litter	%	Transect area covered by leaf litter
	Submerged macrophytes	%	Transect area covered by submerged macrophytes
	Helophytes	%	Transect area covered by helophytes
	Floating macrophytes	%	Transect area covered by floating macrophytes
	Amphibian plants	%	Transect area covered by amphibian plants
	Mud	%	Transect area covered by muddy substrate
	H_diversity_		Shannon index of habitat diversity (Appendix 1)
	HGM		Hydrogeomorphic type/subtype of ponds: storage type, overflow type, puddle type (Appendix 1)
	Hydroperiod		Water regime: episodic, periodic, semi‐permanent, permanent (Appendix 1)
	Canopy cover	%	Canopy cover over the pond
Physical–chemical parameters	Temperature	°C	Water temperature
	pH	−	Water pH
	EC	μS·cm^−1^	Electric conductivity
	DO	mg·L^−1^	Dissolved oxygen
	O_2_%	%	Oxygen saturation
	RedOx	mV	Oxydation/reduction potential
	Alkalinity	mol·L^−1^	Acid neutralizing capacity
	DOC	mg·L^−1^	Dissolved organic carbon
	TOC	mg·L^−1^	Total organic carbon
	TN	mg·L^−1^	Total nitrogen
	NO_3−_N	mg·L^−1^	Nitrate
	NH_4_‐N	mg·L^−1^	Ammonium
	TP	mg·L^−1^	Total phosphorus
	PO_4_‐P	mg·L^−1^	Phosphate
	SO_4_	mg·L^−1^	Sulphate
	Cl	mg·L^−1^	Chloride
	Ca	mg·L^−1^	Calcium
	Mg	mg·L^−1^	Magnesium
	K	mg·L^−1^	Potassium
	Na	mg·L^−1^	Sodium
	Br	mg·L^−1^	Bromine
	TFe	mg·L^−1^	Total iron
	SAC_156_	1·m^−1^	Spectral absorption coefficient
	Chl‐*a*	μg·L^−1^	Chlorophyll‐*a*
	Pheo	μg·L^−1^	Pheophytin

### 2.3 Environmental and spatial variables

A total of 50 variables were measured, including spatial information, land‐use category in the ponds' surroundings, pond habitat, and water physical–chemical parameters (Table 1). Material and methods regarding these variables are provided in the appendices (Appendix 1), with the distribution of each variable (Appendix 2).

### 2.4 Statistical analysis

#### 2.4.1 Alpha‐diversity

Alpha‐diversity is defined here as the taxonomic richness within an individual pond. We used a Bayesian approach to estimate α‐diversity in (1) the three land‐use categories (arable fields, grasslands, forests) and (2) the four crops (barley, corn, rapeseed, and wheat). We used uniform priors and the Gaussian family. Models were run using four Markov chains, 5000 total iterations per chain, including a 1000 iterations burn‐in. Analyses were performed with the probabilistic programming language Stan, using the R package “brms” (Bürkner, 2017). Density and trace plots are in Appendix 3.

#### 2.4.2 Beta‐diversity

Prior to β‐diversity analyses, the absence of spatial autocorrelation of macroinvertebrate communities was verified with a Mantel test (Appendix 4). Total dissimilarity was computed using presence/absence data and the Sørensen dissimilarity index (*β*_SOR_), a widely used index in ecology in both pairwise and multi‐site calculations of beta diversity (see Baselga & Orme, 2012). First, we separated the turnover and nestedness‐resultant components of taxonomic beta‐diversity and computed the three values of multiple‐site dissimilarities: total dissimilarity (*β*_SOR_), turnover (*β*_SIM_), and nestedness (*β*_NES_), using the R package “betapart” (Baselga et al., 2018). They were calculated for: (1) the overall landscape, (2) within each land‐use category (arable fields, grasslands, and forests), and (3) within each crop type (barley, corn, rapeseed, and wheat). We used the function *beta.sample* which randomly selects a specified number of sites (sites = 4, samples = 100), generating distributions of the multi‐site dissimilarity measures to allow the comparison despite the different sample sizes. Then, pairwise β‐diversity between two communities was computed using the Sørensen dissimilarity index (*β*_SOR_), using the function *betadiver* implemented in the R package “vegan” (Oksanen et al., 2019). For each community, pairwise dissimilarity was computed with each other community and then averaged per community.

#### 2.4.3 Random forest models

We used Random Forest (RF) regression to model and interpret α‐ and β‐diversities in the ponds. RFs are a type of machine learning model capable of detecting complex interactions and nonlinearities in data (Breiman, 2001). The RF method develops many regression trees based on a random selection of data and random selection of variables from the original database. It is a useful method for data sets with a high number of parameters relatively to the number of observations. Feature selection, that is, identification of the most relevant variables for explaining α‐ and β‐diversities was performed using the “VSURF” package applied to the full set of available variables (Genuer et al., 2015). For each response variable (α‐ and β‐diversities), we ran the selection procedure 10 times (Appendix 5), as minor variabilities could occur in the final selection of predictors with VSURF (*ntree* = 5000, *mtry* = *n*_0_/3, with *n*_0_ = initial number of variables, Table 1). The first step consisted of eliminating the irrelevant variables from the dataset. The second step was dedicated to select all variables related to the response for interpretation purpose. Then, the predictors selected by the second step were used to build RF models for α‐ and β‐diversities (*ntree* = 5000, *mtry* = *n*_1_/3, with *n*_1_ = the number of predictors selected by step 2). For each response variable, RF models were run 500 times (R package “randomForest”, Liaw & Wiener, 2002), and the Increased Mean Square Error (IncMSE%) was used for quantifying variable importance. Partial dependence plots (PDPs) were used to visualize the nature of the relationships (i.e., linear, monotonic or more complex) between selected predictors and the response variables while averaging the effects of all other predictors in the RF models (“pdp” package, Greenwell, 2017).

#### 2.4.4 γ‐Diversity analysis

Gamma‐diversity was quantified at different levels: the overall γ‐diversity was defined at the landscape scale (all ponds), within each land‐use category (arable fields, forests, and grasslands) and within each of the four crop types (barley, corn, rapeseed, and wheat). Gamma‐diversity was estimated with the Chao2 index, a nonparametric estimator using the small sample correction to consider the different sample sizes in the different land‐use categories and crop types (Oksanen et al., 2019). Differences between γ‐diversity estimates were considered significant if the 95% confidence intervals (CIs) did not overlap. A sensitivity analysis is provided in Appendix 6.

Throughout the main text, we refer to results for taxonomic richness only, unless stated otherwise. The results related to family richness are presented in Appendix 7.

All the statistical analyses were performed using R software version 3.6.1 (R Development Core Team, 2019). Figures were created with “ggplot2” package in R (Wickham, 2016) and BioRender.com.

## 3 RESULTS

### 3.1 Community composition

A total of 122 macroinvertebrate taxa were collected across the 42 ponds (Dryad dataset, link to be provided), covering 53 taxonomic families belonging to Hydrozoa (*n* = 1 family), Turbellaria (*n* = 1), Bivalvia (*n* = 1), Gastropoda (*n* = 3), Oligochaeta (*n* = 2), Hirudinae (*n* = 3), Crustacea (*n* = 1), Nematoda (*n* = 1), and Insecta [*n* = 40, including Diptera (*n* = 15), Coleoptera (*n* = 7), Heteroptera (*n* = 6), Odonata (*n* = 6), Trichoptera (*n* = 4), and Ephemeroptera (*n* = 2)].

### 3.2 α‐Diversity

**FIGURE 1:**
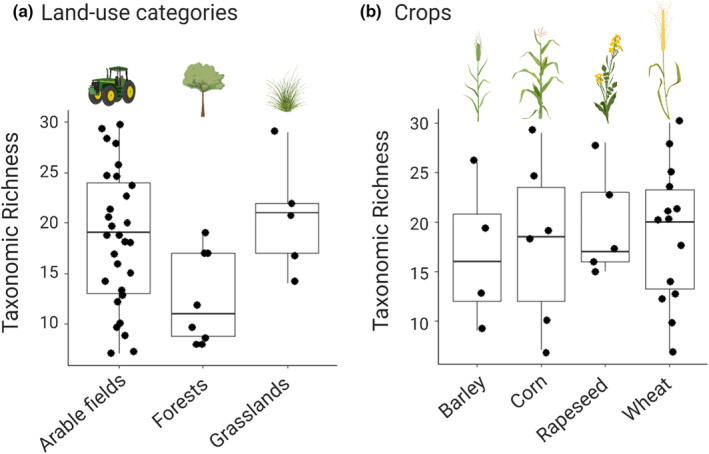
Taxonomic richness of macroinvertebrates recorded from (a) the three different land‐use categories (arable fields, forests, and grasslands) and (b) for the four crops (barley, corn, rapeseed, and wheat). Boxplots show taxonomic richness with min, median, first and third quartiles values, and max. Each ● represents a pond.

**FIGURE 2:**
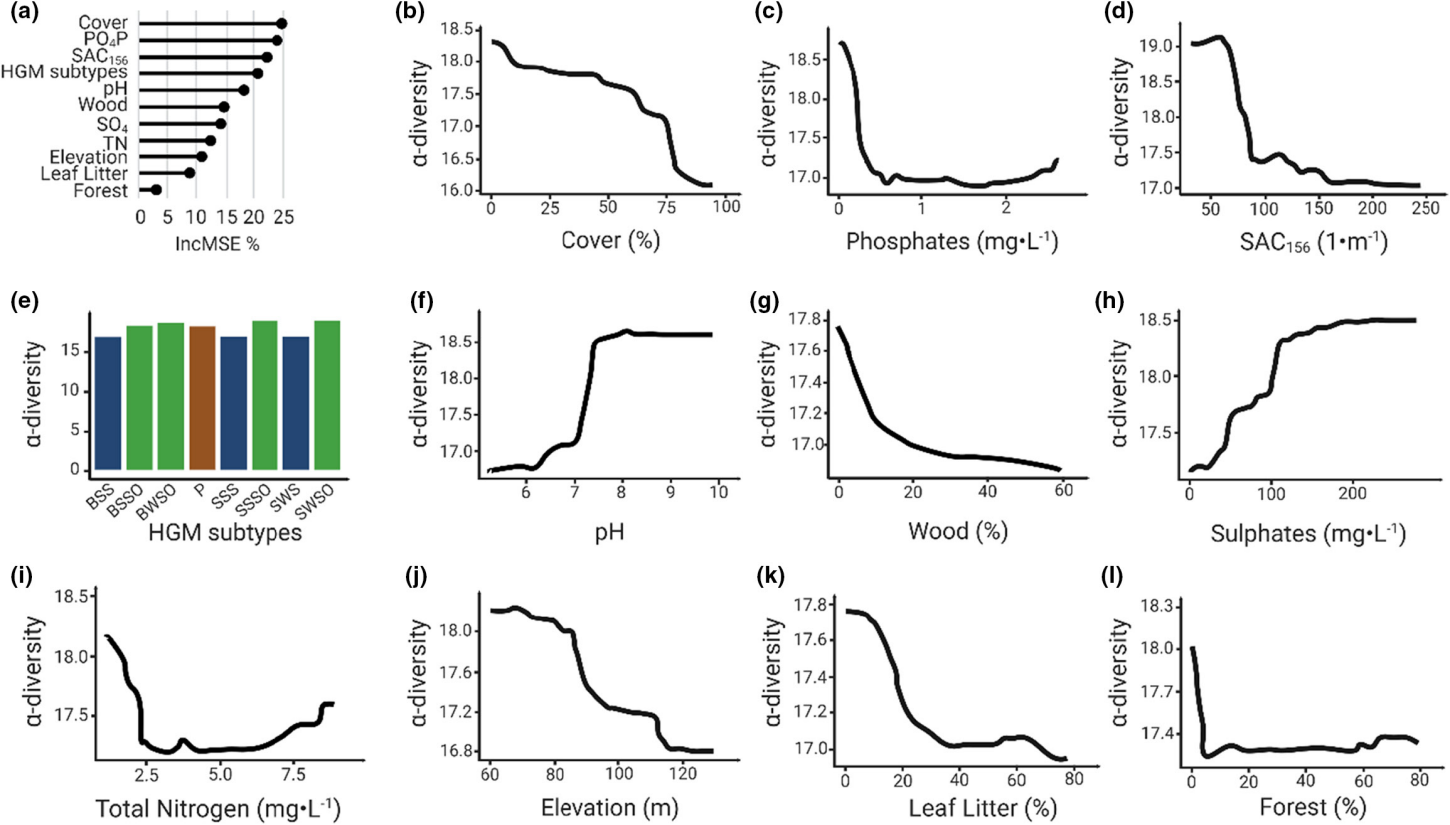
(a) Variable importance (IncMSE%) of the selected variables for interpreting α‐diversity and partial dependence plots showing nonlinear relationships between α‐diversity and each selected variable: (b) canopy cover, (c) phosphate concentration, (d) spectral absorption coefficient, (e) hydrogeomorphic subtypes, (f) pH, (g) woody substrate, (h) sulphate concentration, (i) total nitrogen, (j) elevation, (k) leaf‐litter habitat, and (l) land surface covered by forest. Hydrogeomorphic types are storage type in blue; shore overflow type in green and puddle type in brown (Appendix 1).

Alpha‐diversity of macroinvertebrates was on average 17.6 (± SE 6.6) taxa per pond, ranging from 7 to 30 taxa. It was similar in arable field ponds (posterior mean: 18.5, 95% credibility interval: 16.1–20.9) and grassland ponds (posterior mean difference: 2.0; CI: −4.2 to 8.3), whereas forest ponds had a lower α‐diversity than crop ponds (posterior mean difference: −6.0, CI: −11.2 to −0.9) (Figure 1). Alpha‐diversity was similar among ponds for different crops in adjacent fields: there were on average 16.7 (CI: 9.3–24.1) taxa in barley fields and, compared to that, +1.2 (CI: −8.3 to 10.8) taxa in cornfields, +3.0 (CI: −7.0 to 13.0) taxa in rapeseed fields, and +2.0 (CI: −6.4 to 10.4) in wheat fields (Figure 2). Density and trace plots for both models are provided in Appendix 3.

In total, 11 variables were considered of importance by the VSURF method for interpreting taxonomic α‐diversity of macroinvertebrate communities living in ponds: canopy cover, eutrophication‐related variables (PO_4_‐P, SO_4_, and TN), land‐use (forest %), habitats (wood and leaves), pH, SAC_156_, hydrogeomorphic subtypes, and elevation (Figure 2). Partial dependence scores showed nonlinear effects for all the selected variables, with a high α‐diversity associated with lower canopy cover, lower concentration of nutrients (PO_4_‐P < 0.5 mg·L^−1^ and TN < 2.1 mg·L^−1^), a high concentration of sulphates (SO_4_ > 100 mg·L^−1^), and low proportion of riparian‐vegetation related habitat such as litter and wood. Alpha‐diversity increased with pH until reaching a plateau at high pH value (7.7), and decreased with elevation. Except for elevation, none of the spatial variables was selected among the important variables for interpreting α‐diversity (Appendix 5).

Five variables were considered of importance for interpreting family α‐diversity of macroinvertebrate communities: SAC_156_, SO_4_, PO_4_‐P, wood and forests (see Appendix 7).

### 3.3 Total dissimilarity ( *β*_SOR_ ), turnover ( *β*_SIM_ ), and nestedness ( *β*_NES_ )

**FIGURE 3:**
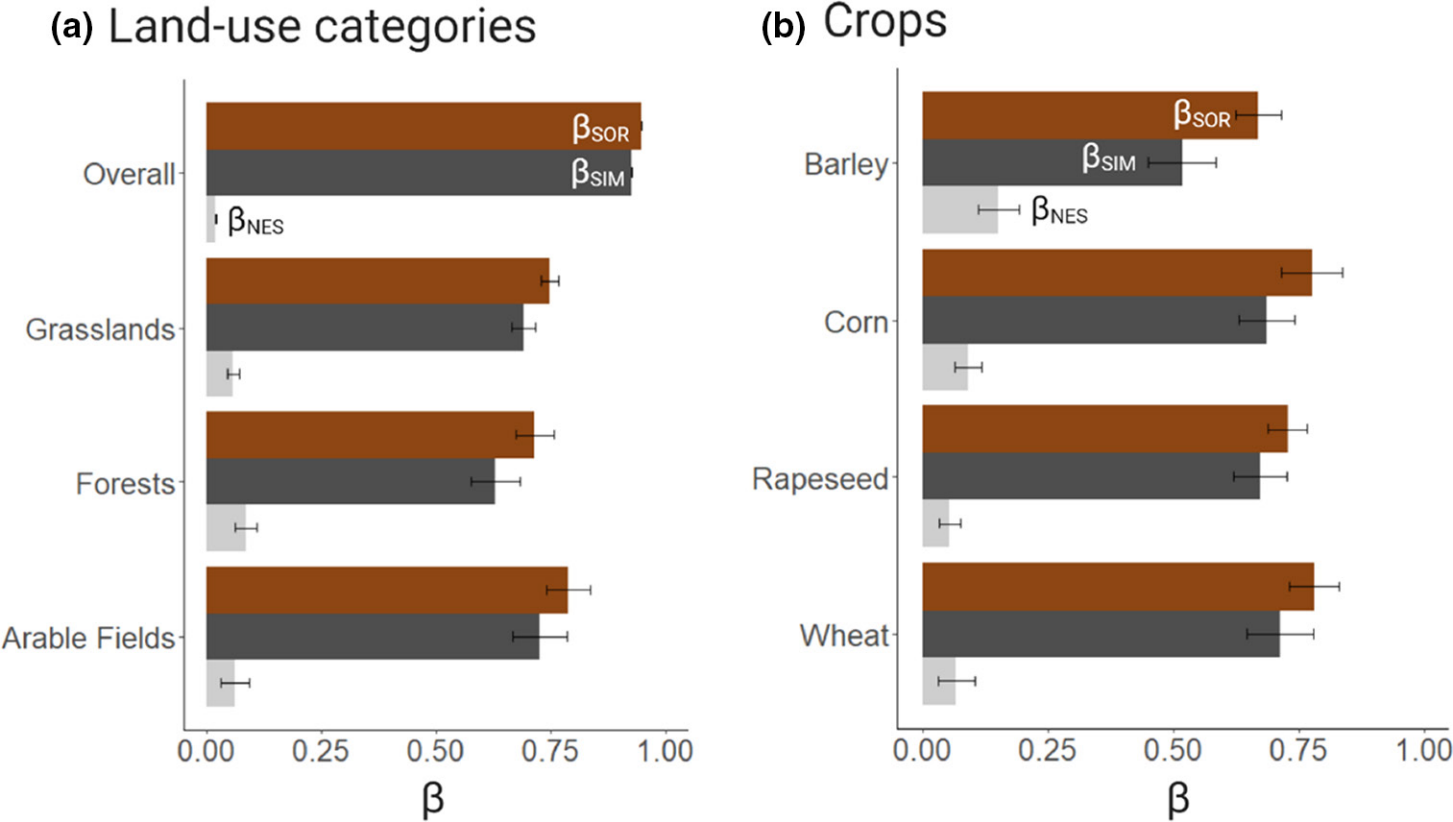
Total Sørensen dissimilarity (*β*_SOR,_ brown) and relative contribution of taxonomic turnover (*β*_SIM_, dark gray) and nestedness (*β*_NES_, light gray) to *β*_SOR_ within (a) the overall landscape for land‐use categories and (b) types of arable fields. The error bars indicate the standard deviation.

Macroinvertebrate communities showed high levels of *β*_SOR_ in the overall landscape (*β*_SOR_ = 0.94). Values of *β*_SOR_ (mean ± SD) were similar among the land‐use categories: 0.79 (±0.04), 0.71 (±0.04), and 0.75 (±0.02) in arable fields, forests, and grasslands, respectively (Figure 3). Most of the variation in macroinvertebrate community composition was driven by spatial turnover in the forest (*β*_SIM_ = 87.9%, *β*_NES_ = 12.1%), the grasslands (*β*_SIM_ = 92.1%, *β*_NES_ = 7.9%), and the arable fields (*β*_SIM_ = 92.1%, *β*_NES_ = 7.9%).

Ponds embedded in the different crops had similar total dissimilarity values: 0.78 (±0.05) in wheat crops, 0.77 (±0.06) in corn crops, 0.73 (±0.04) in rapeseed crops, and 0.67 (±0.05) in barley crops (Figure 3b). In the four crops, spatial turnover explained most of the β‐diversity values: 91.2% in wheat crops, 92.4% in rapeseed crops, 88.3% in a corn crops, and 77.1% in barley crops. A similar pattern was found when *β*_SOR_, *β*_SIM_, and *β*_NES_ were computed at a family level of identification (Appendix 7).

### 3.4 Environmental drivers of *β*_sor_

**FIGURE 4:**
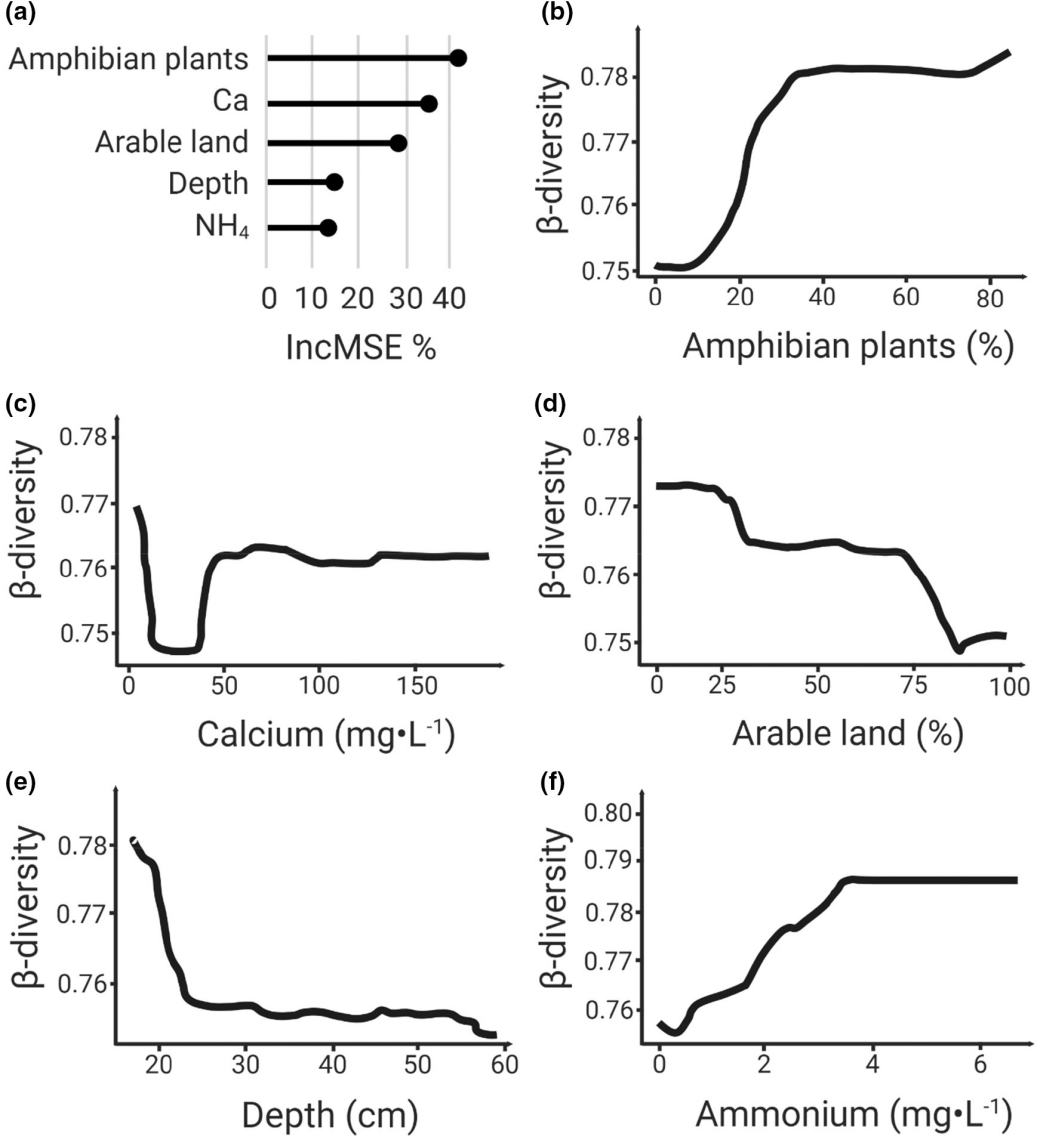
(a) Relative importance (IncMSE%) of selected variables for explaining *β*_SOR_, and partial dependence plots illustrating the relationship between *β*_SOR_ and selected environmental variables: (b) amphibian plants, (c) calcium concentration, (d) the surface of arable land in the surrounding of the pond, (e) depth and (f) ammonium concentration.

Five environmental variables were selected by the VSURF method for explaining taxonomic β‐diversity (Figure 4). High β‐diversity was associated with shallow water (<30 cm), a high proportion of amphibian plants as habitat (>30%), a low proportion of arable land in the adjacent terrestrial surroundings (<25%), and a high concentration of ammonium (>3.2 mg·L^−1^). Calcium was a major driver for β‐diversity, but the relationship between the two is rather complex with a drop in β‐diversity between 8 and 40 mg·L^−1^; above this threshold, β‐diversity is constant. Fewer variables were selected for explaining family β‐diversity: arable land, amphibian plants, and calcium concentration (Appendix 7). None of the spatial variables was selected among the important variables for interpreting β‐diversity (taxonomic and family levels, Appendices 5 and 7).

### 3.5 γ‐Diversity

**FIGURE 5:**
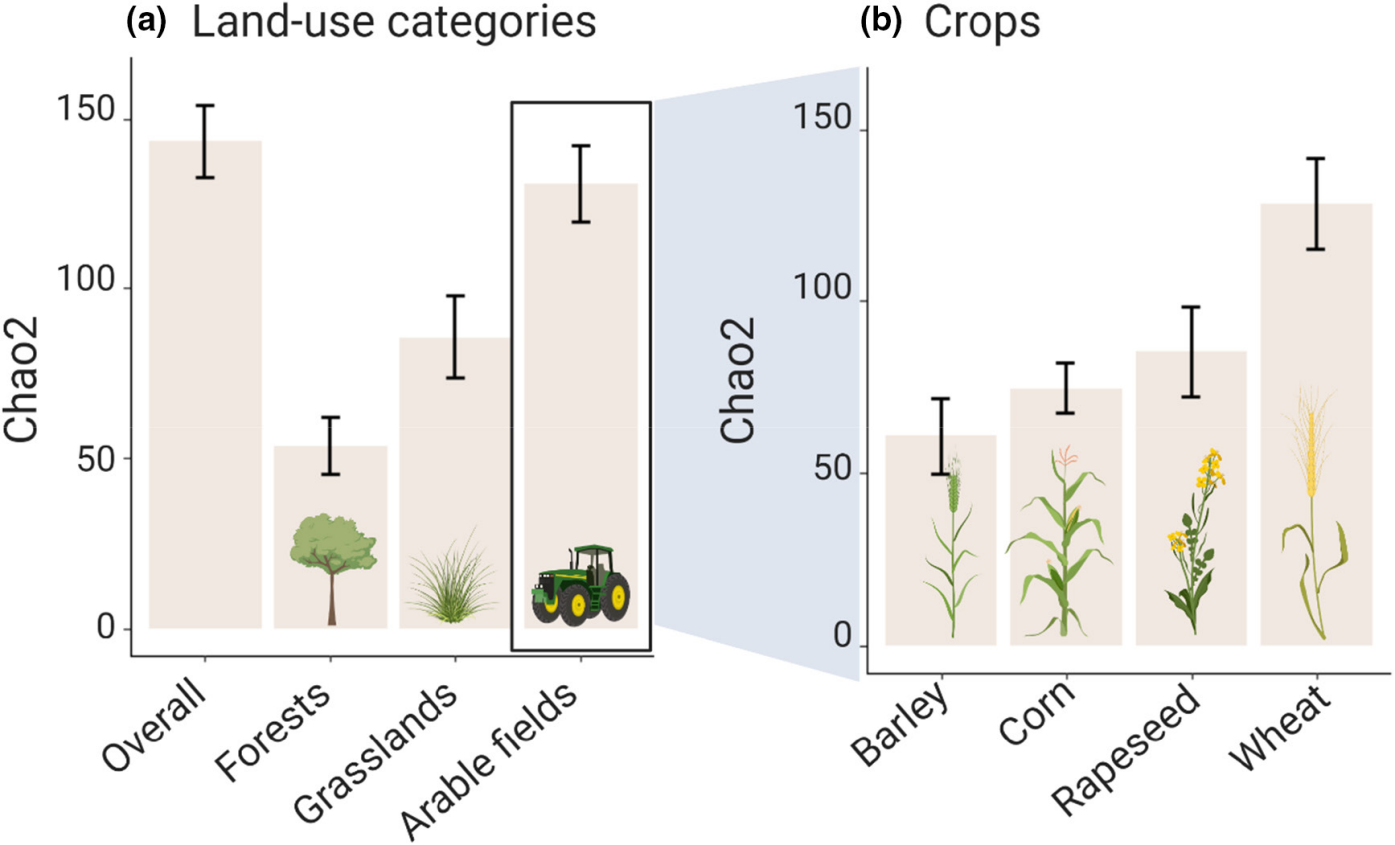
Estimated γ‐diversity (Chao2 estimator ±95% confidence intervals) at (a) the overall landscape level and in the three different land‐use main categories (forests, grasslands, and arable fields) and (b) for four crops in adjacent fields (barley, corn, rapeseed, and wheat).

Estimated γ‐diversity (based on the Chao2 estimator) was higher in arable field ponds than in grassland ponds (arable fields: 130.7 ± 11.2, grasslands: 85.6 ± 12.1) or forest ponds (53.4 ± 8.2, Figure 5a). Among the crops ponds surrounded by wheat crops supported greater macroinvertebrate richness compared to ponds in barley, corn, or rapeseed crops (Figure 5b). The estimated taxonomic γ‐diversity based on bootstrapped Chao2 estimators showed similar patterns (Appendix 6). The estimated family γ‐diversity based on bootstrapped Chao2 estimators was similar between arable fields and grassland ponds, but lower in forest ponds (Appendix 7).

## 4 DISCUSSION

### 4.1 Macroinvertebrate communities shaped by environment rather than by spatial effects

Pond habitat characteristics and terrestrial surroundings (e.g., chemical inputs and riparian vegetation) mainly shaped α‐ and β‐diversities. Neither spatial configuration, distance to the closest pond, nor number of ponds in the surroundings (i.e., proxies of the dispersal and colonization abilities) were selected among the main drivers of taxonomic macroinvertebrate diversity (same results for family diversity, see Appendix 7). These results suggest that macroinvertebrate communities are mainly shaped by environmental variables and that dispersal limitation plays only a small role, which is consistent with previous studies on pond macroinvertebrate assemblages (Heino et al., 2015; Hill et al., 2019). While ditches and channels connecting ponds may provide direct connectivity and migration pathways in modified landscapes, the ponds in our study area are not directly connected via ditches or creeks. Therefore, the main colonization pathways must be active dispersal (flying for terrestrial winged adults) or passive dispersal using vectors such as the abundant animals in the area (e.g., wild boars, foxes, roe deer, waterfowl) or by the wind. The low importance of dispersal limitation for macroinvertebrates at a small scale (220 km^2^, average distance between two ponds: 135 m), and in a long‐term established habitat network (ca. 12,000 years, Kalettka et al., 2001) is not surprising and has been previously reported in this landscape for rotifer communities (Onandia et al., 2021) and in other landscapes for a larger range of taxa (Soininen et al., 2018). Species turnover was the main component of β‐diversity, while nestedness played a smaller role, consistent with previous findings on freshwater pond communities, including macrophytes (Bertuzzi et al., 2019), cladocerans (Viana et al., 2016), and macroinvertebrates (Hill et al., 2017), for which assembly mechanisms were dominated by high species turnover over different geographical scales. The high turnover and dominance of environmental variables driving β‐diversity suggest that niche mechanisms mainly structured our focal communities.

### 4.2 Land‐use and eutrophication

The ponds varied strongly in taxonomic richness, from 7 to 30 taxa per pond. For the ponds in crops, we found a substantially higher number of taxa than in human‐made farm ponds embedded in an agricultural landscape in Southern France (with comparable identification resolution, Céréghino et al., 2008).

Our results also show differences in γ‐diversity between the different crops in adjacent fields. Indeed, while the γ‐diversity of ponds in barley, corn, and rapeseed crops is similar, it is greater in ponds surrounded by wheat crops. The reasons for this pattern are unclear but could be related to supplied chemicals or other differences in the treatment of the different crops.

While these results must be considered for a better understanding of biodiversity in the landscape, RF models showed that the land‐use category and the crop in adjacent fields were not among the main factors explaining either α‐ or β‐diversities (for both taxonomic and family identification levels). Thus, understanding macroinvertebrate biodiversity in a modified landscape should not be restricted to assessing land‐use category patterns but should rather be explored with a broader approach considering the individual effects of agriculturally driven stressors that may act differently at the site scale. The samples in our dataset are biased towards ponds in arable fields, which may affect the inference of our results by inflating β‐diversity due to an imperfect detection of rare species, despite the statistical correction applied (Barwell et al., 2015). Thus, our results have to be interpreted with caution. Yet, they are in line with previous results on pond biodiversity. Ionescu et al. (2022) used environmental DNA and found no difference in taxonomic richness between land‐use types, neither in pond sediment nor in water samples for eukaryotes, *Bacteria* or *Archaea*. Bižić et al. (2022) found changes in the activity of communities (using metatranscriptomics, i.e., full set of expressed genes in a community) depending on land‐use types, but this was not consistent across all sampling campaigns. Taken together, these results suggest the homogenization of freshwater biodiversity most likely resulting from the long‐lasting intensive agriculture. Alpha‐diversity was impaired by nutrient concentrations (total nitrogen and phosphate), highlighting a negative impact of eutrophication in these small freshwater systems. The negative nonlinear relationships between phosphate and total nitrogen concentration on the taxonomic richness of macroinvertebrates suggest deleterious effects of fertilizers. While internal nutrient cycling in ponds is naturally driven by the decay of primary producers and sediment release (Onandia et al., 2018), farming practices are responsible for substantial phosphate and nitrogen enrichments. These farming inputs lead to diffuse nutrient pollution known for impairing freshwater biodiversity (Birk et al., 2020). Nutrient‐rich systems are dominant in the studied pondscape (Kleeberg et al., 2016; Lischeid et al., 2018). While eutrophication is a well‐known cause of biotic impairment, its management in small lowland water bodies is complicated, as eutrophication is often widespread in the landscapes they are embedded in. Furthermore, eutrophication affects taxonomic groups differently (Rosset et al., 2014). Future studies should attempt to quantify threshold values of nutrient and other pollutant concentrations for improving conservation programs.

### 4.3 Roles of vegetation and pond hydrogeomorphology

Terrestrial riparian vegetation surrounding the ponds had a substantial role in shaping α‐diversity. PDPs showed negative and nonlinear relationships between taxonomic richness and each environmental variable related to riparian vegetation (canopy cover, wood, litter, forest, and SAC_156_). Taxonomic richness was lower in heavily shaded ponds with substantial riparian vegetation inputs. This pattern − which has been observed previously in other landscapes or experimental set ups (Batzer et al., 2004; Binckley & Resetarits, 2007; Thornhill et al., 2017)—can be explained either by local species extinction or habitat selection by flying adults. It is known that heavily shaded ponds are usually less colonized by macrophytes due to light limitation. However, these macrophytes represent habitats for macroinvertebrates and valuable oviposition substrates. For species finding suitable niches in shaded ponds, woody habitats (decaying wood, leaf litter, roots, underwater branches, and tree trunks) also represent habitat and egg‐laying sites, as well as food resources—directly or indirectly being the substrate of fungi and algae biofilm (Williams et al., 2018). However, overshaded ponds in intensive agricultural landscapes with substantial water pollution show impoverished biota (Williams et al., 2018). Our β‐diversity results did not show higher or lower β‐diversity in shaded ponds. They are potentially suitable habitats for specialized biota, though, especially when the trees have been established for a long period or in a particular environment, such as temporary ponds full of leaf litter (Williams et al., 2018).

Pond hydrogeomorphic type was identified as a significant determinant of taxonomic richness. Compared to ponds belonging to the storage type, the shore‐overflow and puddle types had a greater taxonomic richness. Due to the nonpermanent shoreline and the inundated surrounding edges for some weeks or months per year, the two latter pond types are causing most conflict about arable land‐use and the periodic crop losses for farmers (Kalettka & Rudat, 2006). Furthermore, the PDPs showed a nonlinear negative relationship between β‐diversity and pond depth, and a nonlinear positive relationship between β‐diversity and coverage of amphibian plants. These results show that more diverse communities of macroinvertebrates inhabit shallow ponds (e.g., shore‐overflow and puddle types) colonized by amphibian plants. In the landscape, these ponds are often temporary, mostly found in arable fields, and usually have a low (or absent) canopy cover. This type of pond, mainly in arable fields, can explain the high turnover value in this land‐use category (92.1% of the β‐diversity). The hydrogeomorphic pond subtypes described by Kalettka and Rudat (2006) are natural geomorphological and ecological gradients in stages of ponds' succession. When the ponds age, they fill up with sediment and organic matter, as a result become smaller and shallower, and then become temporary before turning into solid ground.

### 4.4 Implications for pond conservation and future research

Macroinvertebrates play a crucial role in food‐web dynamics within and beyond freshwater systems. Understanding how human activities such as agriculture affect macroinvertebrate biodiversity in ponds can help conservation policy to protect such small freshwater systems in modified landscapes. Our findings have three main implications for conservation management and future research.

First, macroinvertebrate communities are shaped by environmental rather than by spatial effects, with eutrophication (phosphate and total nitrogen concentrations) impairing macroinvertebrate α‐diversity. Consistent with previous findings on macroinvertebrate communities living in hypertrophic ponds (Rosset et al., 2014), our results highlight the urgent need to reduce nutrient inputs that runoff to the ponds in agricultural landscapes. As responses to eutrophication may change among taxa (Rosset et al., 2014), future research focusing on the identification of their respective threshold values could help to implement conservation programs based on target organisms.

Second, β‐diversity of macroinvertebrate communities is mainly driven by spatial turnover, showing the importance of each pond and the diversity and heterogeneity of habitats they offer in the landscape for supporting regional freshwater biodiversity. This key result shows that effective conservation measures should focus on a large number of ponds in a landscape, not only protecting individual sites inhabited by the highest alpha taxonomic richness.

Third, shallow ponds with a low canopy cover and a high cover of amphibian plants sustain high β‐diversity in the landscape both for macroinvertebrates (this study) and macrophytes (Lozada‐Gobilard et al., 2019). In agricultural landscapes, shallow ponds are highly vulnerable as drought frequency is predicted to increase (Dolgener et al., 2013). Besides climatic threats, these habitats are sometimes integrated into cropland, plowed, and planted in dry seasons/years. Destruction of these habitats has decelerated since the ponds' conservation value has been recognized and protection status been implemented but filling or draining ponds has happened for many decades to gain agricultural land.

## AUTHOR CONTRIBUTIONS

**Alban Sagouis:** Data curation (equal); formal analysis (supporting); investigation (supporting); writing – review and editing (equal). **Camille L. Musseau:** Conceptualization (lead); data curation (equal); formal analysis (lead); investigation (lead); methodology (lead); visualization (lead); writing – original draft (lead); writing – review and editing (equal). **Gabriela Onandia:** Conceptualization (equal); investigation (equal); methodology (equal); resources (equal); writing – review and editing (equal). **Jana S. Petermann:** Conceptualization (equal); funding acquisition (equal); investigation (equal); methodology (equal); project administration (equal); supervision (equal); writing – review and editing (equal). **Jonathan M. Jeschke:** Conceptualization (equal); funding acquisition (equal); investigation (equal); methodology (equal); project administration (lead); writing – review and editing (equal). **Gunnar Lischeid:** Funding acquisition (equal); methodology (equal); project administration (equal); writing – review and editing (equal).

## CONFLICT OF INTEREST

The authors declare no conflict of interest.

## Figures and Tables

**FIGURE A1:**
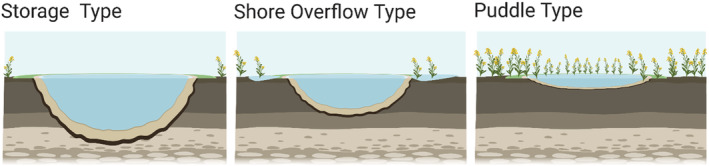
Schematic representation of the three hydrogeomorphologic types of ponds sampled in our study according to Kalettka and Rudat (2006).

**FIGURE A2.1:**
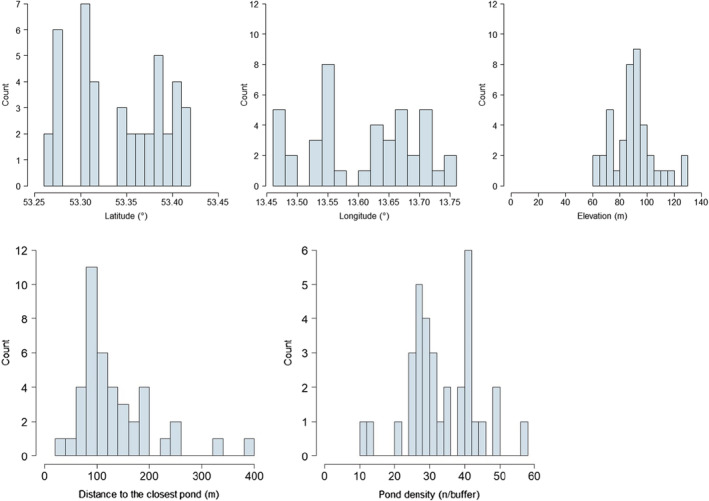
Distribution of the five spatial variables (c.f. Table 1): *Latitude*, *Longitude*, *Elevation*, *Distance to the closest pond*, and *Pond density*.

**FIGURE A2.2:**
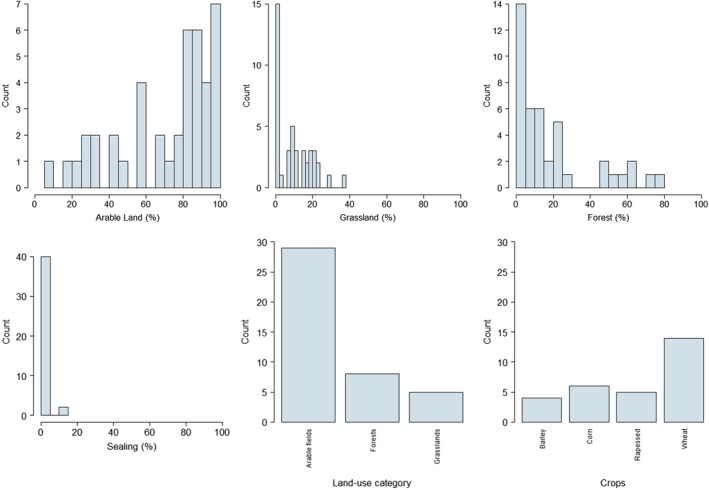
Distribution of the six land‐use: *Arable land*, *Grassland*, *Forest*, *Sealing*, *Land‐use category* and *Crops*.

**FIGURE A2.3:**
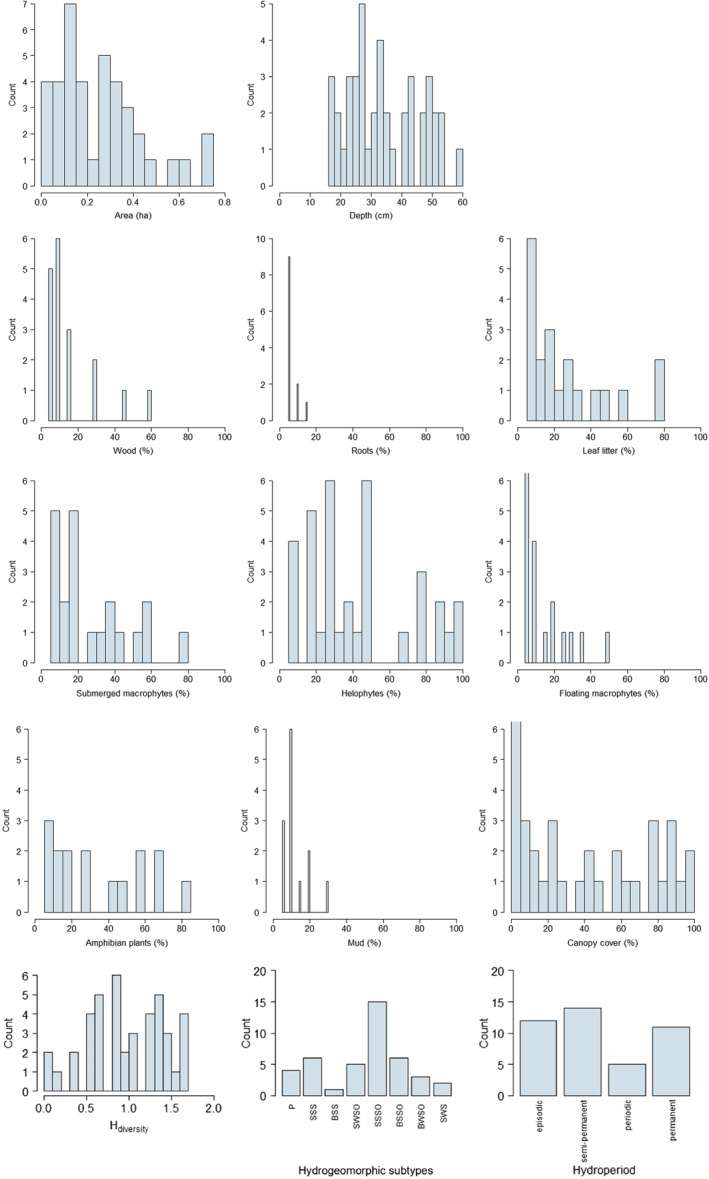
Distribution of the 14 habitat variables: *Surface area*, *Depth*, *Wood*, *Roots*, *Leaf litter*, *Submerged macrophytes*, *Helophytes*, *Floating macrophytes*, *Amphibian plants*, *Mud*, *Canopy cover*, *H*_diversity_, *HGM* (*Hydrogeomorphic subtypes*), and *Hydroperiod*.

**FIGURE A2.4:**
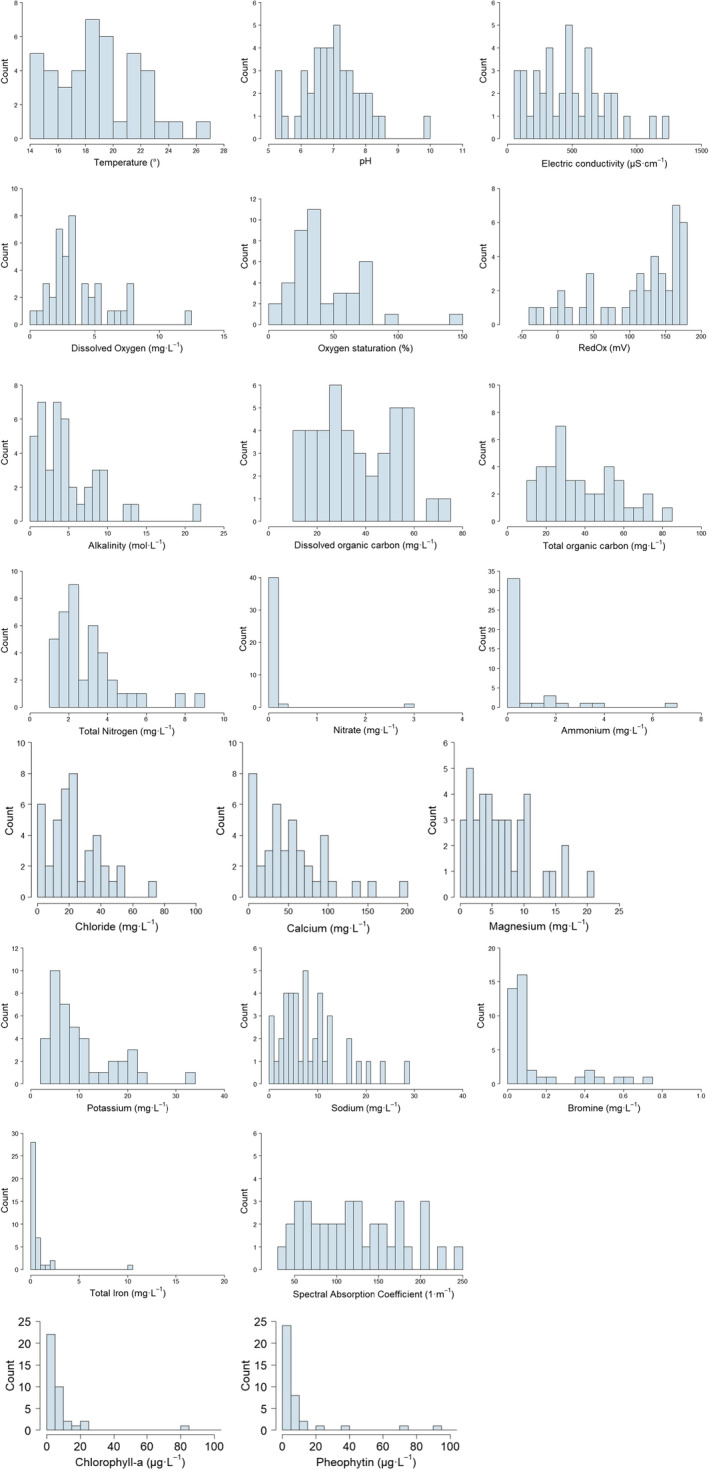
Distribution of the 25 physical‐chemical parameters: *Temperature*, *pH*, Electric conductivity, *Dissolved Oxygen*, *Oxygen saturation*, *RedOx*, *Alkalinity*, *Dissolved organic carbon*, *Total organic carbon*, *Total nitrogen*, *Nitrate*, *Ammonium*, *Total phosphorus*, *Phosphate, Sulphate*, *Chloride*, *Calcium*, *Magnesium*, *Potassium*, *Sodium*, *Bromine*, *Total iron*, *Spectral absorption coefficient*, *Chlorophyall*‐a, *Pheophytin*.

**FIGURE A3.1:**
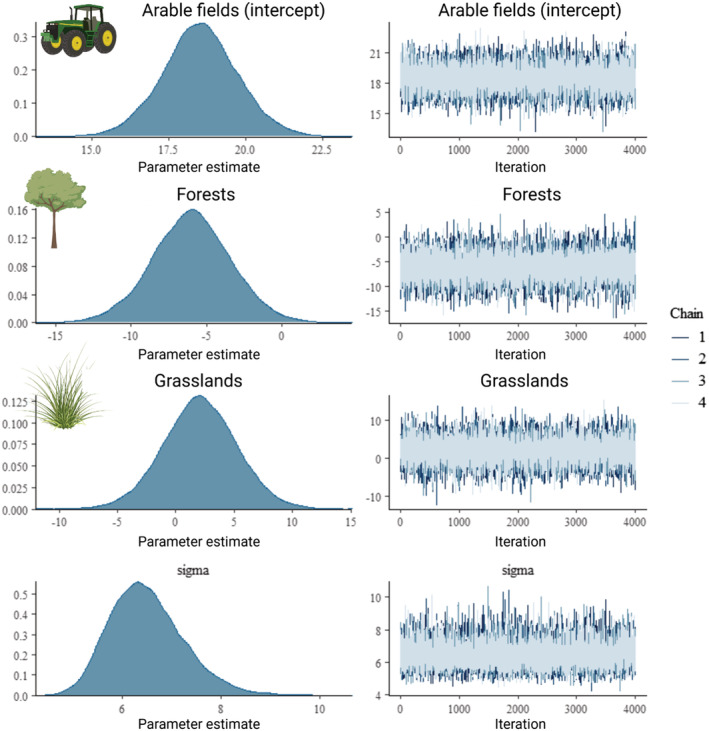
Density plots of the posterior distribution of the model parameters (left panel) and trace plots showing the convergence of the four Markov chains (right panel). This model estimated the posterior mean for α‐diversity (taxonomic richness) in ponds surrounded by arable fields (upper row) and the difference in the forests (second row) and grasslands (third row).

**FIGURE A3.2:**
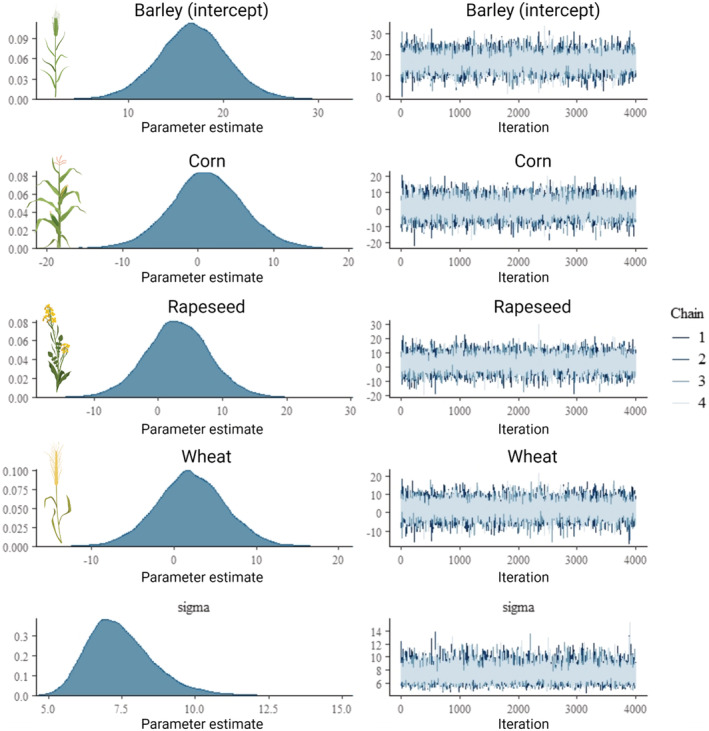
Density plots of the posterior distribution of the model parameters (left panel) and trace plots showing the convergence of the four Markov chains (right panel). This model estimated the posterior mean for α‐diversity (taxonomic richness) in ponds surrounded by different crops: barley (upper row), corn (second row), rapeseed (third row), and wheat (fourth row).

**FIGURE A4.1:**
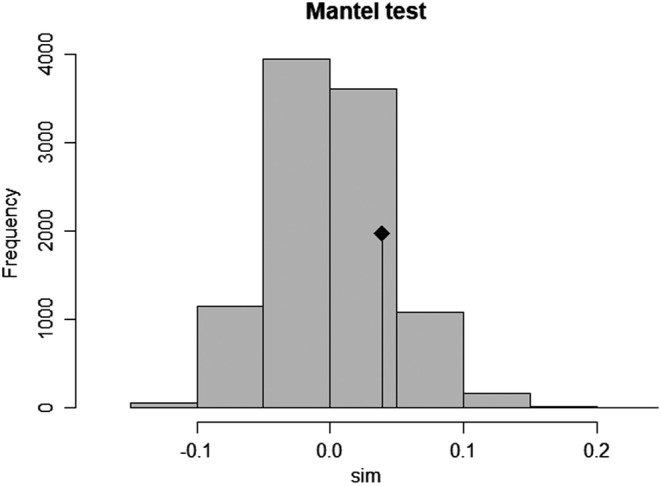
Mantel test's histogram of correlation, the black diamond is the original *r* value, and the grey bars represent the distribution of the 9999 *r* values obtained from permutations.

**FIGURE A4.2:**
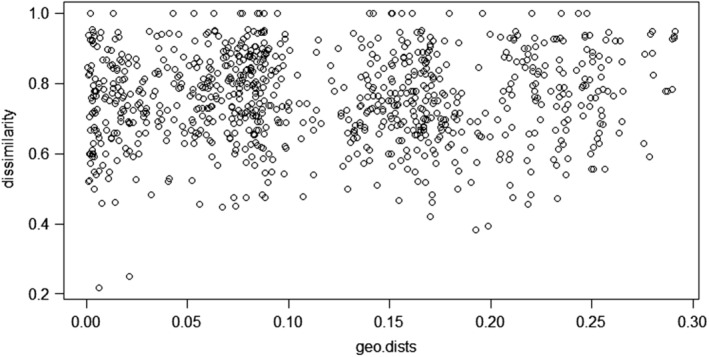
Mantel test scatterplot showing dissimilarity (*y*‐axis) as a function of geographic distance (*x*‐axis) and the absence of correlation.

**FIGURE A6.1:**
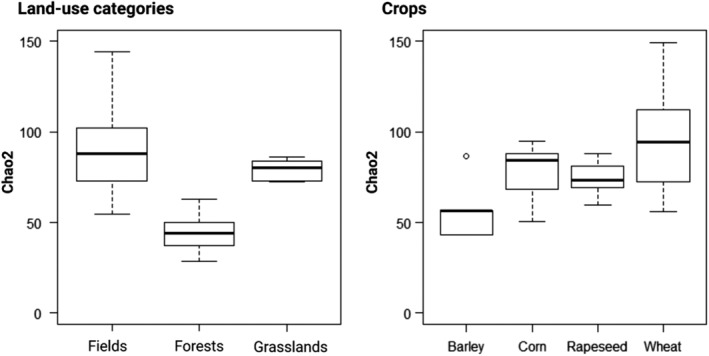
Boxplots showing bootstrapped Chao2 indices distributions (the minimum, the first quartile, the sample median, the third quartile, and the maximum) of macroinvertebrate taxonomic γ‐diversity in the different land‐use categories and the four crops.

**FIGURE A7.1:**
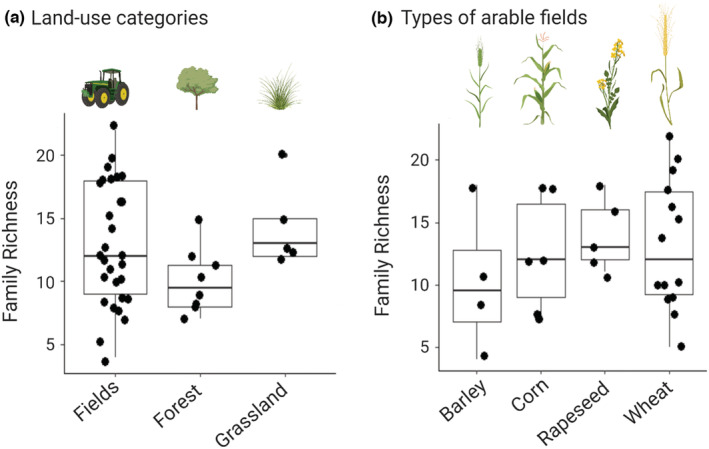
Family richness of macroinvertebrates recorded from (a) the three different land‐use main categories (arable fields, forests, and grasslands) and (b) in the four types of crops in arable fields (barley, corn, rapeseed, and wheat). Box plots show taxonomic richness value for each pond (●), median, first and third quartiles.

**FIGURE A7.2:**
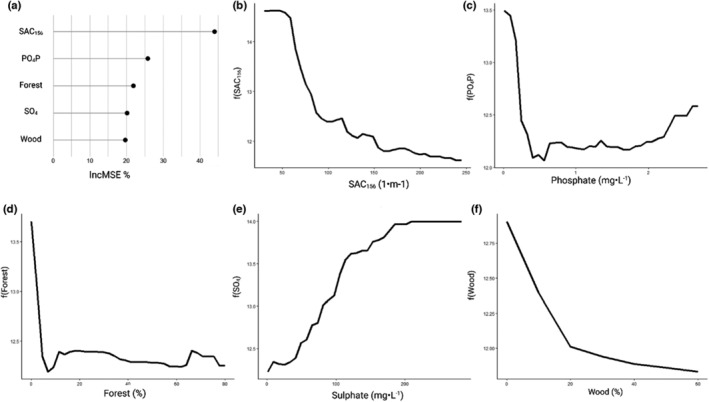
(a) Variable importance scores (IncMSE%) the selected explanatory variables for α‐diversity at family level of identification (a–f) and partial dependence plots showing the relationship between family α‐diversity and each selected variable.

**FIGURE A7.3:**
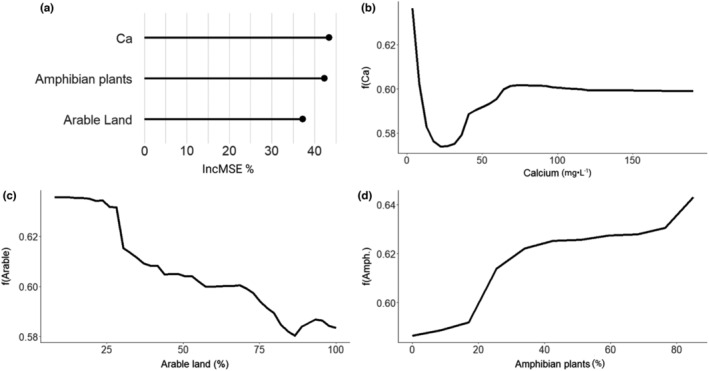
(a) Relative importance (IncMSE%) of selected variables for explaining *β*_SOR_ at family level, (b–d) and partial dependence plots illustrating the relationship between *β*_SOR_ and selected environmental variables.

**FIGURE A7.4:**
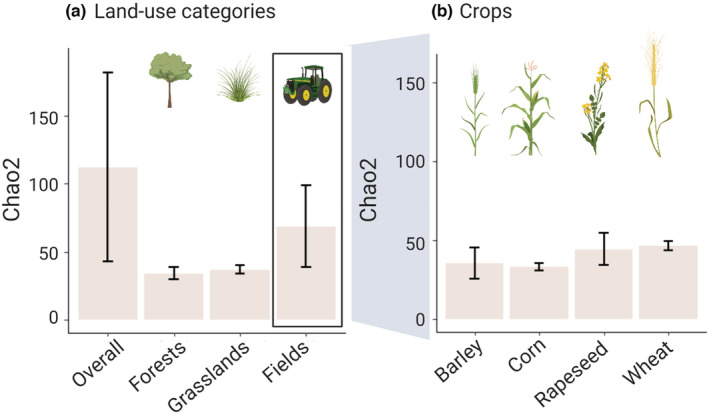
Estimated family γ‐diversity (Chao2 estimator ± 95% confidence intervals) at (a) the overall landscape level and in the three different land‐use categories (forests, grasslands, and arable fields) and (b) in the four crops (barley, corn, rapeseed, and wheat).

**FIGURE A7.5:**
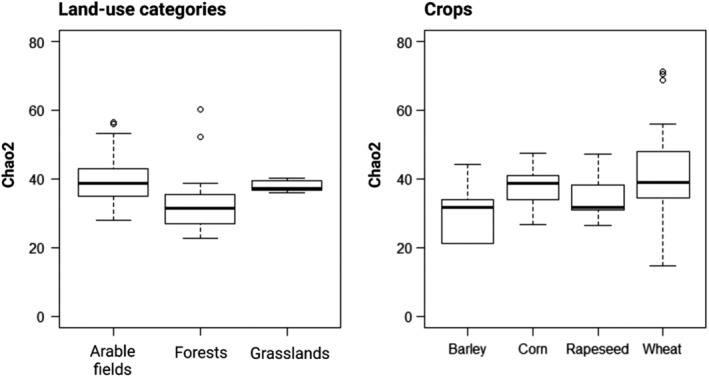
Boxplots showing bootstrapped Chao2 index distributions of macroinvertebrate family γ‐diversity in the different land‐use categories and crops.

## Data Availability

The data supporting the findings of this study is available here: https://doi.org/10.5061/dryad.fj6q573zf.

## APPENDIX 1

### Collection of environmental variables

#### 1 Spatial variables

We collected latitude, longitude (WGS84 coordinate system), and elevation for each pond. Distance to the closest pond and the number of ponds in the surroundings (1 km buffer) were measured using aerial pictures (Google Earth).

#### 2 Land‐use variables

We used CORINE land cover 2012 (https://land.copernicus.eu/pan‐european/corine‐land‐cover/clc‐2012) to determine the land‐use cover around each pond by quantifying the surface (m²) covered by different land‐use categories in a buffer of 1 km from the ponds' centre. The categories were: (1) non‐irrigated arable land, (2) grasslands, (3) forests (including broad‐leaved forest, coniferous forest, and mixed forest), and (4) sealing (farms and roads). The type of crops in which each pond was embedded was recorded in situ during the fieldwork campaign and cross‐validated using the InVeKoS database (Ministry for Infrastructure and Agriculture of the Federal State of Brandenburg).

#### 3 Habitat variables

Habitat was measured and visually estimated at each 1‐m deep‐net sampling transect for macroinvertebrates. Depth was measured with a ruler. Along the transect, we visually estimated the coverage of wood, roots, leaves, submerged macrophytes (*Ceratophyllum submersum* and *C. demersum*), floating macrophytes (*Lemna* spp., *Spirodela* spp.), helophytes (*Phragmites australis*, *Phalaris arundinacea*), and amphibian plants (*Oenanthe aquatica*) (Pätzig et al., 2012). A diversity index of habitats was computed for each pond considering both the richness of sampled habitats and the proportion they represented:

$$H_{\text{diversity}}=-\sum_{i=1}^{h}p_{i}\ln p_{i}$$

where *h* is the number of habitats and *p*_*i*_ the proportion of habitat *i* along the transect.

#### 4 Hydrogeomorphic types

We sampled ponds belonging to three hydrogeomorphic types (Kalettka & Rudat, 2006). First, the storage type, i.e., ponds having sufficient volume for storing incoming water with three subtypes sampled in our study (Figure A1): Big Shallow Storage (BSS), Small Shallow Storage (SSS), and Small Wadable Storage (SWS). Second, the overflow type (Figure A1), i.e., ponds having an insufficient capacity for storing incoming water, resulting in overflow covering pond edges and surrounding terrestrial lands, including four subtypes: Big Shallow SO (BS‐SO), Big Wadable SO (BW‐SO), Small Shallow SO (SS‐SO) and Small Wadable SO (SW‐SO). Finally, the puddle type (P), i.e., small ponds with non‐permanent shore and used as arable land when located in arable fields, particularly during dry seasons (Figure A1). The overflow and puddle types are causing conflicts with land users, as they are responsible for crop losses for some months per year.

#### 5 Hydroperiod

The sampled ponds belong to different hydroperiod categories depending on drought frequency: episodic (long drying up), periodic (annual short drying up), semi‐permanent (drying up every few years), and permanent (no drying up) (Kalettka & Rudat, 2006).

#### 6 Canopy cover

Vegetation on pond edges varied between a few dominant vegetation types: reed, sedges, or riparian trees (mainly *Salix cinerea*, *Alnus glutinosa*, *Betula pubescens*). Canopy cover over the pond was measured using a spherical crown concave densiometer (Concave Model C, Forestry Suppliers, Inc.). Canopy cover was quantified by counting the number of “canopy” dots on a grid lying on a concave mirror reflecting the canopy. Canopy cover was measured in three different locations, with four canopy readings facing each cardinal direction for each pond. The measures were averaged at the location level and then at the site level.

#### 7 Physical‐chemical variables

In total, we collected 25 physical‐chemical water parameters. A set of variables was collected in situ using a multi‐parameter probe (Xylem Analytics Germany Sales GmbH, WTW): water temperature (°C), pH, electric conductivity (EC, μS·cm^−1^), and dissolved oxygen concentration (DO, mg·L^−1^). Alkalinity was measured in situ using a field test set (MSD Sharp & Dohme GmbH). At each pond, water was collected for further water chemistry analysis. Concentrations of sulphate (SO_4_, mg·L^−1^), bromine (Br, mg·L^−1^), nitrate (NO_3_‐N, mg·L^−1^), and chloride (Cl, mg·L^−1^) were determined using ion chromatography with an 882 Compact IC plus (Deutsche Metrohm GmbH & Co. KG). Calcium (Ca, mg·L^−1^), magnesium (Mg, mg·L^−1^), potassium (K, mg·L^−1^), sodium (Na, mg·L^−1^), and total iron (TFe, mg·L^−1^) were analysed using ICP‐OES (ICP‐iCAP 6300 DUO, ThermoFisher SCIENTIFIC GmbH). The samples were analysed using spectrophotometry (SPECORD 210 plus, Analytik Jena AG) for quantifying phosphates (PO_4_‐P, mg·L^−1^), ammonium (NH_4_‐N, mg·L^−1^), and spectral absorption coefficient (SAC_156_, 1 m^−1^). After microwave digestion, total phosphorus (TP, mg·L^−1^) was analysed as soluble phosphorus (Gallery™ Plus, Microgenics GmbH). Dissolved organic carbon (DOC, mg·L^−1^), total organic carbon (TOC, mg·L^−1^), and total nitrogen (TN, mg·L^−1^) were quantified using elemental analysis with chemiluminescence detection (TOC‐Vcph, Shimadzu Deutschland GmbH).

Chlorophyll‐*a* (Chl‐*a*, μg·L^−1^) and pheophytin (Pheo, μg·L^−1^) were quantified as proxies of phytoplankton biomass and therefore primary production. Before chlorophyll‐*a* (Chl‐*a*) and pheophytin (Pheo) analysis, water samples were filtered through a 100 μm mesh to remove larger detritus and organisms. Pigment concentrations were determined from samples collected onto glass‐fibre filters (GF/F, Cytiva Europe GmbH) that were immediately placed inside a glass vessel and stored at −80°C in the dark until they were processed. Chl‐*a* and Pheo were extracted with 96% ethanol and measured spectrophotometrically (DIN 38 412‐16, 1985).

## APPENDIX 2

### Distribution of spatial and environmental variables

Figures A2.1, A2.2, A2.3 and A.2.4 show the distribution of the spatial and environmental variables.

#### Habitat variables

Physical‐chemical parameters

## APPENDIX 3

### Bayesian models: density and trace plots

Figures A3.1 and A3.2 show the density plots of the posterior distribution of the model parameters and trace plots showing the convergence of the four Markov chains for the estimation of α‐diversity in the different land‐use categories and types of crops, respectively.

## APPENDIX 4

### Test for spatial autocorrelation

Spatial autocorrelation: the correlation between the spatial distribution of ponds and their communities, was tested before statistical analyses using a Mantel test (R package “ade4,” Dray & Dufour, 2007). The Mantel test is a method testing for correlation between matrices, here: a distance matrix computed on pond coordinates (*geo.dists*) and the dissimilarity matrix computed on pond invertebrate communities (*dissimilarity*) (Figure A4.2). The Mantel statistic (*r*) was calculated as the correlation between the two original matrices, then matrix values were randomly permuted, and the same statistic was calculated under each permutation (*n* = 9999) and compared to the original statistic (Figure A4.1).

The results show no correlation between the distance matrix *geo.dists* and the dissimilarity matrix (*r* = .04, *p*‐value = .018, Figure A4.2) and therefore that there is no significant spatial autocorrelation among the sampled communities.

## APPENDIX 5

### Variable selection for random forest models

The variables included in the random forest analyses were selected using the “VSURF” package (Genuer et al., 2015). As minor variability may occur in selecting variables with VSURF, the procedure was carried out 10 times. We report here the outcomes for the 10 selection procedures. The two tables below report the list of the 50 variables used in the selection, and the “x” identifies the selected variable at the Threshold step (i.e., eliminating the variables of minor importance) and at the “Interpretation step” (i.e., select all variables related to the response for interpretation purpose) for the 10 selection procedures. All variables selected for the second step were at least once included in the pool of variables used for the RF models. (TABLES A5.1 and A5.2).

#### 1 Alpha‐diversity

**TABLE A5.1:** List of variables selected by the VSURF procedures (repeated 10 times) at the Threshold step and Interpretation step for explaining the taxonomic α‐diversity of macroinvertebrates living in the ponds.

Group	Variable	Threshold										Interpretation									
Run		1	2	3	4	5	6	7	8	9	10	1	2	3	4	5	6	7	8	9	10
Spatial	Latitude																				
	Longitude																				
	Elevation	●	●	●	●	●	●	●	●	●	●	●	●	●	●	●	●	●	●	●	●
	Closest pond																				
	Pond density																				
Land‐use	Arable land		●																		
	Grasslands																				
	Forests	●	●	●	●	●	●	●	●	●	●	●	●	●	●	●	●	●	●	●	●
	Sealed land																				
	Land‐use categories																				
	Crops																				
Habitat	Surface area																				

Depth																				

Wood	●	●	●	●	●	●	●	●	●	●	●	●	●	●	●	●	●	●	●	●

Roots																				

Leaves litter	●	●	●	●	●	●	●	●	●	●		●	●	●	●	●	●	●	●	●

Submerged macrophytes																				

Helophytes																				

Floating macrophytes																				

Amphibian plants		●																		

Mud																				

*H*_diversity_																				

HGM	●	●	●	●	●	●	●	●	●	●	●	●	●	●	●	●	●	●	●	●

Hydroperiod																				

Canopy cover	●	●	●	●	●	●	●	●	●	●	●	●	●	●	●	●	●	●	●	●
Physical‐chemical parameters	Temperature																				
	pH	●	●	●	●	●	●	●	●	●	●	●	●	●	●	●	●	●	●	●	●
	EC	●	●	●	●	●	●	●	●	●	●										
	DO																				
	O_2_%																				
	RedOx																				
	Alkalinity		●					●													
	DOC	●	●	●	●	●	●	●	●	●	●										
	TOC	●	●	●	●	●	●	●	●	●	●										
	TN	●	●	●	●	●	●	●	●	●	●	●	●	●	●	●	●	●	●	●	●
	NO_3_‐N																				
	NH_4_‐N																				
	TP	●	●	●	●	●	●	●	●	●	●										
	PO_4_‐P	●	●	●	●	●	●	●	●	●	●	●	●	●	●	●	●	●	●	●	●
	SO_4_	●	●	●	●	●	●	●	●	●	●	●	●	●	●	●	●	●	●	●	●
	Cl																				
	Ca																				
	Mg		●	●																	
	K		●	●	●			●	●	●	●										
	Na							●	●	●											
	Br																				
	TFe	●	●	●	●	●	●	●	●	●	●										
	SAC_156_	●	●	●	●	●	●	●	●	●	●	●	●	●	●	●	●	●	●	●	●
	Chl‐*a*	●	●	●	●	●	●	●	●	●	●										
	Pheo	●	●	●	●	●	●	●	●	●	●										

#### 2 Beta‐diversity

**TABLE A5.2:** List of variables selected by the VSURF procedures (repeated 10 times) at the Threshold step and Interpretation step for explaining the taxonomic β‐diversity of macroinvertebrates living in the ponds.

Group	Variable	Threshold										Interpretation									
Run		1	2	3	4	5	6	7	8	9	10	1	2	3	4	5	6	7	8	9	10
Spatial	Latitude																				
	Longitude																				
	Elevation																				
	Closest pond																				
	Pond density																				
Land‐use	Arable land	●	●	●	●	●	●	●	●	●	●	●	●	●	●	●	●	●	●	●	●
	Grasslands																				
	Forests				●	●	●														
	Sealed land																				
	Land‐use categories																				
	Crops																				
Habitat	Surface area																				
	Depth	●	●	●	●	●	●	●	●	●	●			●			●				
	Wood																				
	Roots																				
	Leaf litter																				
	Submerged macrophytes																				
	Helophytes																				
	Floating macrophytes																				
	Amphibian plants	●	●	●	●	●	●	●	●	●	●	●	●	●	●	●	●	●	●	●	●
	Mud																				
	*H*_diversity_																				
	HGM																				
	Hydroperiod																				
	Canopy cover																				
Physical‐chemical parameters	Temperature																				
	pH																				
	EC																				
	DO																				
	O_2_%																				
	RedOx																				
	Alkalinity																				
	DOC																				
	TOC																				
	TN																				
	NO_3_‐N																				
	NH_4_‐N	●	●	●	●	●	●	●	●	●	●	●	●	●	●	●	●	●	●	●	●
	TP																				
	PO_4_‐P																				
	SO_4_																				
	Cl																				
	Ca	●	●	●	●	●	●	●	●	●	●	●	●	●	●	●	●	●	●	●	●
	Mg	●	●	●	●	●	●	●	●	●	●										
	K																				
	Na																				
	Br																				
	TFe																				
	SAC_156_							●	●	●	●										
	Chl‐*a*	●	●	●	●	●	●	●	●	●	●										
	Pheo																				

## APPENDIX 6

### Sensitivity analyses for γ‐diversity

The sample sizes differed among land‐use categories and types of arable fields. Therefore, we ran two types of analyses to ensure that estimated γ‐diversity values were not affected by this methodological bias.

First, the Chao2 estimator was computed using the dedicated function for small samples correction (Oksanen et al., 2019). The outcomes are reported in the Results section of the manuscript.

Second, we compared the values of Chao2 among land‐use categories and types of arable fields using a bootstrap (*n*_iteration_ = 50) method. 50 Chao2 estimators were calculated for each group based on a minimal common number *n*−1 (*n* being the number of ponds in the group with the smallest number of sites). Therefore, the Chao2 estimators were calculated with a minimal common number of 4 and 3 for land‐use categories and types of arable fields, respectively. We used a Bayesian approach to estimate Chao2 in (1) the three land‐use categories (arable fields, grasslands, forests) and (2) the four types of arable fields (barley, corn, rapeseed, and wheat). We used uniform priors and the Gaussian family, and models were run using four Markov chains, 5000 total iterations per chain, including a 1000‐iteration burn‐in. Analyses were performed with the probabilistic programming language Stan, using the R package “brms” (Bürkner, 2017).

For the estimated γ‐diversity among land‐use categories, the bootstrapped communities showed similar trends as the results presented in the main manuscript. The Chao2 estimator was higher for arable field ponds (88.1, 95% CI: 84.4–92.0) than for grassland (−9.2, 95% CI: −14.5 to −3.9) or forest ponds (−43.1, 95% CI: −48.3 to −37.8) (Figure A6.1).

Within the arable field category, the lowest bootstrapped Chao2 estimator was found for barley fields (58.9, 95% CI: −14.5 to −3.9), intermediate values of Chao2 were found for corn fields (+19.2, 95% CI: 10.5–27.8) and rapeseed fields (+15.9, 95% CI: 7.4–24.5), and the highest Chao2 was found wheat fields (+40.3, 95% CI: 31.8–48.9) (Figure A6.1).

## APPENDIX 7

### Analyses at the family level

#### 1 α‐diversity

On average, ponds were inhabited by 12.4 (±4.4) macroinvertebrate families, ranging from 4 to 22 families in the sampled ponds. Using family richness instead of taxonomic richness (i.e., richness encompassing taxa with different levels of identification, see main manuscript), we found similar patterns. However, family richness in arable field ponds (posterior mean: 12.8, 95% credibility interval: 11.2–14.5) did not significantly differ from grassland (posterior mean difference: 1.6; CI: −2.6 to 5.9) and forest ponds (posterior mean difference: −2.8, CI: −6.4 to 0.7, Figure A7.1).

Family richness was similar among ponds embedded in different types of arable fields: there were on average 10.2 (CI: 5.0–15.2) families in barley fields and compared to that +2.3 (CI: −4.33 to 9.0) families in cornfields, +3.8 (CI: −3.0 to 10.7) families in rapeseed fields, and +3.0 (CI: −2.8 to 8.9) families in wheat fields (Figure A7.1), showing similar trends as the taxonomic richness results.

**TABLE A7.1:** List of variables selected by the VSURF procedures (repeated 10 times) at the Threshold step and Interpretation step for explaining the family α‐diversity of macroinvertebrates living in the ponds.

Group	Variable	Threshold										Interpretation									
Run		1	2	3	4	5	6	7	8	9	10	1	2	3	4	5	6	7	8	9	10
Spatial	Latitude																				
	Longitude																				
	Elevation	●	●	●	●	●	●	●	●	●	●										
	Closest pond																				
	Pond density																				
Land‐use	Arable land																				
	Grasslands																				
	Forests	●	●	●	●	●	●	●	●	●	●	●	●	●	●	●	●	●	●	●	●
	Sealed land																				
	Land‐use categories																				
	Crops																				
Habitat	Surface area																				
	Depth																				
	Wood	●	●	●	●	●	●	●	●	●	●	●	●	●	●	●	●	●	●	●	●
	Roots																				
	Leaf litter	●	●	●	●	●	●	●	●	●	●										
	Submerged macrophytes									●											
	Helophytes																				
	Floating macrophytes																				
	Amphibian plants																				
	Mud																				
	*H*_diversity_	●	●	●	●	●	●	●	●	●	●										
	HGM																				
	Hydroperiod																				
	Canopy cover	●	●	●	●	●	●	●	●	●	●										
Physical‐chemical parameters	Temperature																				
	pH	●	●	●	●	●	●	●	●	●	●										
	EC																				
	DO																				
	O_2_%																				
	RedOx																				
	Alkalinity																				
	DOC	●	●	●	●	●	●	●	●	●	●										
	TOC	●	●	●	●	●	●	●	●	●	●										
	TN	●	●	●	●	●	●	●	●	●	●										
	NO_3_‐N																				
	NH_4_‐N																				
	TP	●	●	●	●	●	●	●	●	●	●										
	PO_4_‐P	●	●	●	●	●	●	●	●	●	●	●	●	●	●	●	●	●	●	●	●
	SO_4_	●	●	●	●	●	●	●	●	●	●	●	●	●	●	●	●	●	●	●	●
	Cl																				
	Ca	●	●	●	●	●	●	●	●	●	●										
	Mg																				
	K																				
	Na																				
	Br																				
	TFe																				
	SAC_156_	●	●	●	●	●	●	●	●	●	●	●	●	●	●	●	●	●	●	●	●
	Chl‐*a*																				
	Pheo	●	●	●	●	●	●	●	●	●	●										

Five variables were considered of importance for interpreting family α‐diversity of macroinvertebrate communities: SAC_156,_ SO_4_, PO_4_‐P, wood and forests (TABLE A7.1). As for taxonomic richness reported in the main manuscript, partial dependence scores showed nonlinear effects of all the selected variables, with a high family α‐diversity associated with low concentrations of SAC_156_ and PO_4_‐P, low proportion of wood in the pond and low proportion of forests in the surroundings (Figure A7.2).

#### 2 Total dissimilarity (*β*_SOR_), turnover (*β*_SIM_), and nestedness (*β*_NES_)

Family β‐diversity results showed the same patterns as the results of β‐diversity quantified at the taxonomic level and reported in the main manuscript. We found no difference of total dissimilarity (*β*_SOR_) between communities living in the different land‐use categories and among the types of crops (TABLE A7.2). Furthermore, the relative contributions of family turnover and family nestedness displayed the same patterns as the taxonomic turnover and taxonomic nestedness. However, taxonomic turnover had higher percentages in the arable fields than family turnover. (TABLE A7.3)

**TABLE A7.2:** Total Sørensen dissimilarity (*β*_SOR_), turnover (*β*_SIM_) and nestedness (*β*_SIM_) and relative contributions (%) of *β*_SIM_ and *β*_SIM_ to *β*_SOR_ within the overall landscape, land‐use categories, and types of arable fields.

	*β*_SOR_	*β*_SIM_	*β*_NES_	% *β*_SIM_	% *β*_NES_
Land use					
Overall	0.93 (±0.00)	0.89 (±0.00)	0.03 (±0.00)	96.3	3.7
Forest	0.67 (±0.05)	0.59 (±0.07)	0.07 (±0.03)	88.7	11.3
Grassland	0.64 (±0.02)	0.57 (±0.03)	0.07 (±0.02)	89.3	10.7
Arable fields	0.65 (±0.06)	0.53 (±0.08)	0.12 (±0.05)	81.2	18.5
Crops					
Barley	0.59 (±0.05)	0.30 (±0.05)	0.28 (±0.07)	52.3	47.7
Corn	0.63 (±0.13)	0.52 (±0.14)	0.11 (±0.03)	82.0	18.0
Rapeseed	0.56 (±0.06)	0.48 (±0.08)	0.08 (±0.02)	85.4	14.6
Wheat	0.65 (±0.07)	0.49 (±0.12)	0.15 (±0.09)	75.6	24.4

#### 3 Environmental drivers of *β*_SOR_

**TABLE A7.3:** List of variables selected by the VSURF procedures (repeated 10 times) at the Threshold step and Interpretation step for explaining the family β‐diversity of macroinvertebrates living in the ponds

Group	Variable	Threshold										Interpretation									
Run		1	2	3	4	5	6	7	8	9	10	1	2	3	4	5	6	7	8	9	10
Spatial	Latitude	●																			
	Longitude	●	●	●	●	●	●	●	●	●	●										
	Elevation																				
	Closest pond																				
	Pond density																				
Land‐use	Arable land	●	●	●	●	●	●	●	●	●	●	●	●	●	●	●	●	●	●	●	●
	Grasslands	●	●	●	●	●	●	●	●	●	●										
	Forests																				
	Sealed land	●	●	●	●	●	●	●	●	●	●										
	Land‐use categories																				
	Crops	●	●	●	●	●	●	●	●	●	●										
Habitat	Surface area																				
	Depth																				
	Wood	●	●	●	●	●	●	●	●	●	●										
	Roots	●			●	●		●													
	Leaf litter																				
	Submerged macrophytes																				
	Helophytes							●		●											
	Floating macrophytes																				
	Amphibian plants	●	●	●	●	●	●	●	●	●	●	●	●	●	●	●	●	●	●	●	●
	Mud																				
	*H*_diversity_																				
	HGM																				
	Hydroperiod																				
	Canopy cover																				
Physical‐chemical parameters	Temperature																				
	pH	●	●	●	●	●	●	●	●	●	●										
	EC																				
	DO																				
	O_2_%																				
	RedOx																				
	Alkalinity																				
	DOC																				
	TOC																				
	TN																				
	NO_3_‐N																				
	NH_4_‐N	●	●	●	●	●	●	●	●	●	●										
	TP																				
	PO_4_‐P																				
	SO_4_	●	●	●	●	●	●	●	●	●	●										
	Cl																				
	Ca	●	●	●	●	●	●	●	●	●	●	●	●	●	●	●	●	●	●	●	●
	Mg																				
	K	●	●	●	●	●	●	●	●	●	●										
	Na																				
	Br							●	●		●										
	TFe																				
	SAC_156_																				
	Chl‐*a*	●	●	●	●	●	●	●	●	●	●										
	Pheo																				

Two variables were considered of importance for interpreting family β‐diversity of macroinvertebrate communities: arable land, calcium concentration and amphibian plants (TABLE A7.2, Figure A7.3).

#### 4 γ‐diversity

Estimated γ‐diversity at the family level (based on the Chao2 estimator) was higher in arable field ponds than in grassland (arable fields: 130.7 ± 11.2 and grasslands: 85.6 ± 12.1) or forest ponds (53.4 ± 8.2, Figure A7.4A). Within the arable field category, wheat ponds supported greater macroinvertebrate richness than ponds in fields sown with barley, corn, and rapeseed (Figure A7.4B). There was no significant difference in γ‐diversity among barley, corn, and rapeseed field ponds (Figure A7.4B).

To deal with differences in sample sizes, we conducted the same sensitivity analyses as the one explained in Appendix 6, but at the family level of identification. (Figure A7.5).

The bootstrapped communities showed different patterns than the results obtained when working at the taxonomic level of identification presented in the manuscript. The family Chao2 estimator was similar between arable field ponds (39.6, 95% CI: 37.8–41.5) and grassland ponds (−1.5, 95% CI: −4.2 to 1.1) and forests had the lowest family γ‐diversity (−6.40, 95% CI: −9.1 to −3.8).

Within the arable field category, we found differences between the bootstrapped family Chao2 estimator of communities living in the ponds embedded in different types of fields: like the results from the taxonomic level of identification, the lowest Chao2 estimator was found for barley fields (31.5, 95% CI: 28.5–34.5), and was similar to the Chao2 estimator for rapeseed fields (+3.1, 95% CI: −1.1 to 7.39). It was higher for corn fields (+5.9, 95% CI: 1.7–10.1) and highest for wheat fields (+12.4, 95% CI: 8.2–16.5).
